# Defining in vivo dose‐response curves for kidney DNA adduct formation of aristolochic acid I in rat, mouse and human by an in vitro and physiologically based kinetic modeling approach

**DOI:** 10.1002/jat.4024

**Published:** 2020-07-07

**Authors:** Rozaini Abdullah, Sebastiaan Wesseling, Bert Spenkelink, Jochem Louisse, Ans Punt, Ivonne M.C.M. Rietjens

**Affiliations:** ^1^ Division of Toxicology Wageningen University Wageningen The Netherlands; ^2^ Department of Environmental & Occupational Health, Faculty of Medicine and Health Sciences Universiti Putra Malaysia Selangor Malaysia

**Keywords:** aristolochic acid I (AAI), DNA adduct formation, in vitro‐in vivo extrapolation, physiologically based kinetic (PBK) modeling, reverse dosimetry

## Abstract

Aristolochic acid I (AAI) is a well‐known genotoxic kidney carcinogen. Metabolic conversion of AAI into the DNA‐reactive aristolactam‐nitrenium ion is involved in the mode of action of tumor formation. This study aims to predict in vivo AAI‐DNA adduct formation in the kidney of rat, mouse and human by translating the in vitro concentration‐response curves for AAI‐DNA adduct formation to the in vivo situation using physiologically based kinetic (PBK) modeling‐based reverse dosimetry. DNA adduct formation in kidney proximal tubular LLC‐PK1 cells exposed to AAI was quantified by liquid chromatography‐electrospray ionization‐tandem mass spectrometry. Subsequently, the in vitro concentration‐response curves were converted to predicted in vivo dose‐response curves in rat, mouse and human kidney using PBK models. Results obtained revealed a dose‐dependent increase in AAI‐DNA adduct formation in the rat, mouse and human kidney and the predicted DNA adduct levels were generally within an order of magnitude compared with values reported in the literature. It is concluded that the combined in vitro PBK modeling approach provides a novel way to define in vivo dose‐response curves for kidney DNA adduct formation in rat, mouse and human and contributes to the reduction, refinement and replacement of animal testing.

## Abstract 2

This study describes a physiologically based kinetic (PBK) modeling‐based reverse dosimetry approach to convert in vitro concentration‐response curves for aristolochic acid I‐DNA adduct formation quantified in LLC‐PK1 cells to in vivo dose‐response curves in the kidney of rat, mouse and human. The predicted DNA adduct levels were within an order of magnitude from values reported in the literature. In conclusion, the in vitro PBK approach predicts DNA adduct formation with a similar level of uncertainty, as observed in the experimental animal studies.

## 1 INTRODUCTION

The development of science‐based nonanimal testing strategies in the safety assessment of chemicals in humans is an important challenge. Current efforts in this area focus on the development and use of in vitro alternative testing strategies using cells in culture resulting in concentration‐response curves. However, concentration‐response curves from in vitro models are of limited use for risk and safety assessments in humans, because the risk assessment requires in vivo dose‐response curves from which points of departure can be derived. A novel alternative testing strategy that can be used to solve this discrepancy between in vitro and in vivo data involves the translation of in vitro concentration‐response curves to in vivo dose‐response curves using physiologically based kinetic (PBK) modeling‐based reverse dosimetry (Abdullah, Alhusainy, Woutersen, Rietjens, & Punt, 2016; Chen, Peijnenburg, de Haan, & Rietjens, 2019; DeJongh, Nordin‐Andersson, Ploeger, & Forsby, 1999; Louisse et al., 2010; Suparmi et al., 2019). By using this integrated in vitro‐in silico approach, in vivo dose‐response levels and points of departure for risk assessment can be defined based on in vitro concentration‐response curves. Previously, we reported proofs of principle for this approach, including the prediction of in vivo DNA adduct formation of alkenylbenzenes (Punt et al., 2016) or α,β‐unsaturated aldehydes (Kiwamoto, Rietjens, & Punt, 2012; Kiwamoto, Spenkelink, Rietjens, & Punt, 2013) and the prediction of in vivo developmental toxicity of tebuconazole (Li et al., 2017), glycol ethers (Louisse et al., 2010), phenol (Strikwold, Spenkelink, Woutersen, Rietjens, & Punt, 2013) and retinoic acid (Louisse, Bosgra, Blaauboer, Rietjens, & Verwei, 2014). In our previous study, we translated in vitro concentration‐response curves for cytotoxicity of aristolochic acid I (AAI) in LLC‐PK1 or MDCK cells to in vivo dose‐response curves for kidney toxicity from which we derived BMDL_10_ values (benchmark dose 10% lower confidence limit) that can be used as points of departure for risk assessment (Abdullah et al., 2016). Given that the ultimate critical effect of AAI toxicity is not only kidney toxicity but also DNA adduct formation resulting in AAI‐induced mutagenesis and carcinogenesis, the aim of the present study was to translate in vitro concentration‐response curves for DNA adduct formation in a kidney cell line to in vivo dose‐response curves for DNA adduct formation in the kidney of rat, mouse and human. Over the past years, a number of in vivo studies has been carried out to evaluate the dose‐dependent DNA adduct formation in the kidney of rats (Bieler et al., 1997; Chan et al., 2008; Dong et al., 2006; Mei, Arlt, Phillips, Heflich, & Chen, 2006; Pfau, Schmeiser, & Wiessler, 1990a) and mice (Arlt et al., 2011; Shibutani et al., 2007; Yun et al., 2012) exposed to AAI or a mixture of AAs. In addition, human studies on AA kidney DNA adduct formation in patients with AA nephropathy (AAN) are available (Bieler et al., 1997; Nortier et al., 2000), enabling validation of the predictions made.

AAs are the main components in all *Aristolochia* species that have been used as a traditional medicine to treat arthritis, gout, rheumatism and snake bites (Arlt, Stiborova, & Schmeiser, 2002; Frei, Würgler, Juon, Hall, & Graf, 1985). However, products containing AAs were prohibited after Mengs and colleagues discovered the carcinogenic effects of AAs in rats (Mengs, 1983; Mengs, Lang, & Poch, 1982). The risks of exposure to AAs became even more evident in 1993 when more than 1800 Belgian women were accidentally exposed to AAs via slimming pills (Vanherweghem et al., 1993) and later, more than 100 of these young women developed chronic kidney failure, developing into cancer of the kidneys and the urinary tract in several patients (Vanhaelen, Vanhaelen‐Fastre, But, & Vanherweghem, 1994; Vanherweghem et al., 1993). These medical disorders were associated with the presence of kidney AA‐DNA adducts (Schmeiser, Bieler, Wiessler, de Strihou, & Cosyns, 1996). A large body of evidence suggests that AA‐induced DNA adduct formation, followed by cellular proliferation and fixation of mutations, is responsible for cancer development in AA‐treated animals (Arlt et al., 2002; Arlt et al., 2007) and humans (Arlt et al., 2002; Nortier & Vanherweghem, 2002).

**FIGURE 1:**
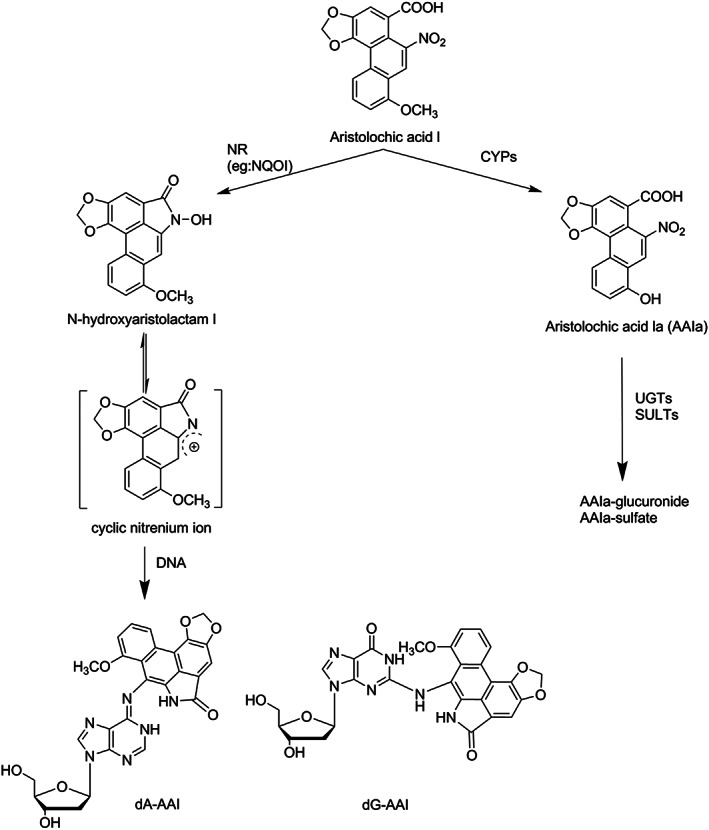
Metabolic pathways for detoxification, bioactivation and DNA adduct formation of AAI. AAI, aristolochic acid I; CYPs, cytochromes P450; dA‐AAI, deoxyadenosine AAI; dG‐AAI, deoxyguanosine AAI; NQOI, NAD(P)H:quinone oxidoreductase; NR, nitroreduction; SULTs, sulfotransferases; UGTs, uridine 5′‐diphospho‐glucuronosyltransferases

AAs are nitrophenanthrene carboxylic acids and the most studied congeners are 8‐methoxy‐6‐nitrophenanthro‐(3,4‐*d*)‐1,3‐dioxolo‐5‐carboxylic acid (AAI) and its 8‐demethoxylated form (AAII) (Kumar, Prasad, & Parmar, 2003). It was found by Schmeiser and coworkers, that not only mixtures of AAs were found to be carcinogenic but that AAI alone could induce tumors in rats (Schmeiser et al., 1990). AAI is the major component in the mixtures of AAs (Schmeiser et al., 1996; Stiborová et al., 2003). Formation of the *N*‐hydroxyaristolactam metabolite of AAI is considered to lead to DNA adduct formation, whereas formation of AAIa is considered as a detoxification reaction (Figure 1) (Shibutani et al., 2010). Nitroreduction of AAI leads to the formation of *N*‐hydroxyaristolactams, which is catalyzed by both cytosolic and microsomal enzymes of which NAD(P)H:quinone oxidoreductase is the most important enzyme (Chen et al., 2011; Jadot, Anne‐Emilie, Joëlle, & Nathalie, 2017; Stiborová et al., 2003; Stiborová, Frei, Arlt, & Schmeiser, 2008; Stiborová et al., 2011). This reaction generates a reactive nitrenium intermediate, which can bind to DNA, resulting in the formation of AAI‐DNA adducts (Pfau et al., 1990a; Pfau, Schmeiser, & Wiessler, 1990b) (Figure 1). The major adduct formed is 7‐(deoxyadenosin‐*N* ^6^‐yl)aristolactam I (deoxyadenosine‐AAI, dA‐AAI), which has also been found to be the most persistent adduct in the kidney tissue (Arlt et al., 2002; Bieler et al., 1997). Although AAI may be bioactivated in other organs such as the liver, the kidney has been shown to be the major target organ for AAI‐induced toxicity including AAI‐DNA adduct formation and tumor induction (Mei et al., 2006). This tissue‐specific toxicity has been suggested to be because the capacity of the DNA repair processes in the kidney is lower than in other organs (Schmeiser, Schoepe, & Wiessler, 1988) and/or to the ability of the proximal tubule cells to take up and concentrate AAs and their metabolites, making the kidney more susceptible to AA‐induced toxicity (Mei et al., 2006).

As indicated, the aim of the present study was to predict in vivo AAI‐DNA adduct formation in the kidney of rat, mouse and human by extrapolation of in vitro concentration‐response curves for AAI‐DNA adduct formation to the in vivo situation using PBK modeling‐based reverse dosimetry. By defining dose‐response curves for rat, mouse and human, using only in vitro and in silico methods, the outcome of this study may provide new insights in alternative methods for human risk assessment, particularly with respect to possible species‐dependent differences in dose‐dependent DNA adduct formation and related carcinogenicity.

## 2 MATERIAL AND METHODS

### 2.1 Chemicals

AAI was purchased from Sigma‐Aldrich. The LLC‐PK1 porcine cell line (ATCC® CL‐101™) was obtained from the American Type Culture Collection (ATCC). Dulbecco's modified Eagle medium (DMEM), phosphate‐buffered saline and trypsin‐EDTA were purchased from Gibco and fetal calf serum from Lonza BioWhittaker. dA, deoxyguanosine (dG), *N,N*‐dimethylformamide, zinc powder, phosphodiesterase I from *Crotalus adamanteus* (venom phosphodiesterase), phosphodiesterase II from bovine spleen (spleen phosphodiesterase), nucleus PI and alkaline phosphatase were purchased from Sigma‐Aldrich. Dimethyl sulfoxide (>99.9%) was obtained from Acros Organics. Acetonitrile (ACN; ULC/MS grade) was obtained from Biosolve BV. Formic acid and ethanol were obtained from VWR Merck.

### 2.2 General outline for physiologically based kinetic modeling‐based reverse dosimetry approach

Development of the in vitro PBK approach to predict in vivo dose‐response curves for DNA adduct formation consisted of the following steps: (i) establishment of in vitro concentration‐response curves for AAI‐dependent DNA adduct formation in the LLC‐PK1 cell line; (ii) translation of the in vitro concentration‐response curves into in vivo dose‐response curves for DNA adduct formation in rat, mouse and human using established PBK models (Abdullah et al., 2016) describing in vivo kinetics of AAI in rat, mouse and human; and (iii) evaluation of the predictions against available in vivo data.

### 2.3 In vitro DNA adduct formation in LLC‐PK1 cells

The LLC‐PK1 cell line was cultured in 75 cm^2^ flasks at 37°C in a humidified atmosphere of 5% CO_2_ in DMEM supplemented with fetal calf serum (10% v/v). Cells were subcultured three times a week, using 1% (v/v) trypsin‐EDTA to detach the cells. Cytotoxicity was evaluated using the MTT assay as previously described (Abdullah et al., 2016).

In total, approximately 1 × 10^6^ cells/flask were seeded. At confluency of 80%‐90%, the cells were exposed for 24 hours to AAI at different concentrations ranging from 0.5 to 20 μm (final concentration in the DMEM without serum) added from 200 times concentrated stock solutions in dimethyl sulfoxide. In line with previous studies (Romanov, Sidorenko, Rosenquist, Whyard, & Grollman, 2012; Sidorenko et al., 2014), exposure of cells to AAI was performed in serum‐free medium to prevent binding of AAI to serum proteins, which would decrease the free concentration of AAI to which the cells were exposed (Dickman, Sweet, Bonala, Ray, & Wu, 2011).

To obtain a sufficient amount of DNA, all concentrations of AAI were tested in duplicate and the duplicate samples were pooled. After the exposure to AAI, cells were scraped in 5 mL phosphate‐buffered saline, collected in a 10 mL tube and centrifuged at 417 g for 5 minutes. The pellets were stored at −20°C until DNA isolation. For DNA isolation, a QIAamp DNA Mini Kit from Qiagen was applied according to the procedure as recommended by the supplier. The yield and purity of the extracted DNA were determined using Nanodrop 1000 technology by measuring the absorbance ratio A260/280 nm. DNA samples with an absorbance ratio of 1.8‐2.0 were considered pure. Digestion of DNA was performed as previously described (Paini et al., 2010) with minor modifications. In short, 40 μL P1 buffer (300 mm sodium acetate, 1 mm ZnSO_4_, pH 5.3), 20 μL spleen phosphodiesterase solution (0.001 U/μL) and 10 μL nuclease PI (0.5 U/μL in water) were added to 50 μg DNA and incubated for 4 hours at 37°C. Then, 40 μL PA buffer (500 mm Tris, 1 mm EDTA, pH 8.0), 20 μL venom phosphodiesterase solution (0.0002 U/μL in water), and 15 μL alkaline phosphatase (0.27 U/μL) were added and the sample was incubated for another 2 hours at 37°C. The hydrolyzed samples were evaporated to dryness and reconstituted in 50 μL water. The samples were kept at –80°C until analysis using liquid chromatography‐electrospray ionization‐tandem mass spectrometry (LC‐ESI‐MS/MS).

### 2.4 Synthesis of deoxyadenosine‐aristolochic acid I and deoxyguanosine‐ristolochic acid I adducts

The synthesis of the dA‐AAI and dG‐AAI adducts was performed by reaction of AAI with dA or dG using a modification of the protocol described previously (Yun et al., 2012). In short, 100 μL of AAI in *N,N*‐dimethylformamide (10 mm) was mixed with 80 mg of preactivated zinc dust (<150 μm, 99.95%). Then, 1000 μL of dA or dG dissolved in potassium phosphate (50 mm, pH 5.8) were added to the AAI/zinc dust mixture to give a final concentration range that varied from 0 to 100 μm. After incubation in the dark at 37°C for 16 hours, the samples were put on ice for 30 minutes and centrifuged at 41 700 g for 10 minutes. Previously, synthesis of dA‐AAI by this procedure was reported to result in 2% yield of the adduct (Yun et al., 2012).

In the present study the synthesized dA‐ and dG‐AAI adducts were purified on a Waters HPLC system using a GRACE Alltima C18 column (150 mm 5 μm). The Waters system consists of a Waters 600 Controller and Pump, a 717plus autosampler and a 2996 DAD. The eluent used was linearly changed from 100% nanopure H_2_O to 100% ACN in 25 minutes. The fractions containing the adducts were collected (dA‐AAI between 17 and 18 minutes; dG‐AAI between 14 and 15 minutes). Fractions collected from multiple runs were pooled, and the fractions were freeze dried after evaporation of the ACN under a stream of N_2_. After that the purified adducts were analyzed and quantified by LC‐MS performed as described further below. Quantification was based on the peak intensity of the neutral fragment loss of 116 *m*/*z* in dA‐AAI (543➔427) and dG‐AAI (559➔443) as compared with a calibration curve made for these fragmentations using dA and dG. This was possible because the fragmentation pattern of the ribonucleoside moiety of dA and dG was similar being also 116 *m*/*z* for dA (252➔136) and dG (268➔152). Using this quantification method, the dA‐AAI and dG‐AAI synthesis was estimated to have an efficiency of 0.04% and 1.47%, respectively.

The synthesized adduct samples obtained were used in LC‐ESI‐MS/MS analyses to define a calibration curve for the quantification of dA‐AAI and dG‐AAI adducts in the cell studies.

### 2.5 Liquid chromatography‐electrospray ionization‐tandem mass spectrometry method for detection and quantification of deoxyadenosine‐aristolochic acid I and deoxyguanosine‐aristolochic acid I

The LC‐ESI‐MS/MS method for the detection and quantification of dA‐AAI and dG‐AAI was adapted from Yun et al. (2012). LC‐ESI‐MS/MS analysis was performed on a 200 series high‐performance liquid chromatography system (Perkin Elmer) coupled to an API 3000 system (Applied Biosystems) as previously described (Paini et al., 2012; Punt et al., 2007). In brief, 10 μL of sample was injected on a Zorbax Extend‐C18 column (Agilent), 2.1 × 50 nm, 3.5 μm 80 Å, with a Zorbax guard column. A gradient was made with ultrapure water containing 0.1% formic acid as solvent A and 100% ACN as solvent B. The flow rate was set to 0.3 mL/min. In a total run of 15.5 minutes, the starting condition was 90:10 (A/B) for 1 minute followed by changing to 50:50 in 2.5 minutes, then to 0:100 in 1 minute and remaining at 0:100 for another 2 minutes before returning to the starting condition over 1 minute and keeping these conditions for 8 minutes to allow the column to re‐equilibrate at room temperature.

The MS analysis in the positive ion mode was optimized with the following settings: nebulizer gas (air) at 10 psi, curtain gas (nitrogen) at 10 psi, ion spray voltage at 4000 V, collision energy at 28 eV, ion source temperature at 400°C, declustering potential set at 69 V, focusing potential at 175 V, entrance potential at 13 V, and collision cell exit at 15 V. Nitrogen was used as sheath gas turbo, ion spray, with a pressure of 7000 L/h. The dwell time per transition was 0.05 seconds. A divert valve was used to discard the gradient after elution of the peak. The MS was operated in MRM mode with the following *m*/*z* transitions; 543➔427 for dA‐AAI and 559➔443 for dG‐AAI.

Data analysis of the calibration series and the samples was performed using the Analyst software version 1.5 (Applied Biosystem). Calibration curves were derived by plotting the peak area of synthesized dA‐AAI or dG‐AAI against the concentration of dA‐AAI or dG‐AAI and were used to determine the amount of DNA adducts in the samples of AAI‐exposed cells. The amount of dA‐AAI or dG‐AAI detected in the samples was related to the total amount of digested DNA detected in each sample and adjusted for the mass conversion of double strands DNA per 1000 nucleotides (nt) that correspond to 607.6 g/mol, to quantify the number of adducts per 10^8^ nt.

### 2.6 Physiologically based kinetic models for rat, mouse and human

In our previous work (Abdullah et al., 2016), PBK models were developed that describe the toxicokinetics of AAI in rat, mouse and human. In the present work, the same PBK models were used to convert concentrations to dose levels that would induce the DNA adduct levels observed in vitro. To this purpose an equation describing the AAI concentration‐dependent DNA adduct formation in LLC‐PK1 kidney cells in vitro was added to the kidney compartment of the PBK model. In this way, the kinetic parameters for bioactivation of AAI to its DNA adduct forming metabolite were implicitly included in the combined in vitro‐in silico model, as this takes place in the LLC‐PK1 cells in vitro.

The set of differential equations describing the mass balance equations can be found in Data S1A (see Supporting Information). The PBK model equations were solved with Berkeley Madonna (version 8.3.18; UC Berkeley) using Rosenbrock's algorithms for solving stiff systems. A sensitivity analysis was performed to evaluate the influential parameters on the model output. Normalized sensitivity coefficients were calculated for the area under the curve (AUC) of the AAI venous blood concentration in the kidney as the model output (*C*) using the following equation:

(1) $$\mathrm{SC}=\left(C\prime -C\right)/\left(P^{\prime }-P\right)\times \left(P/C\right)$$

where *C* is the initial value of the model output, *C*′ is the modified value after changing parameter value *P*, *P* is the initial parameter value and *P*′ is the modified parameter value (Evans & Andersen, 2000). A 5% increase in parameter values was chosen to analyze the effect of a change in a parameter. The sensitivity analysis was conducted for oral exposure to single doses of 0.1 and 100 mg/kg body weight of AAI to simulate the influences of low‐ and high‐dose levels to the model output.

### 2.7 Translation of in vitro concentration‐response curves to in vivo dose‐response curves

Based on the in vitro concentration‐response curve for AAI‐DNA adduct formation in LLC‐PK1 kidney cells, the in vivo dose‐response curves for DNA adduct formation in the kidney of rat, mouse and human were predicted by PBK modeling‐based reverse dosimetry. To this end, the concentration‐response data from the in vitro DNA adduct formation experiment, were translated to AUC‐response data by multiplying the concentration with the exposure time (24 hours). The extrapolation of the in vitro free AUC‐response curve to the in vivo situation was done by assessing which oral doses are required in the PBK model to reach equivalent free AUC values of the AAI venous blood in the kidney as the conventional marker of the biological active concentration in a tissue that can be linked to toxicodynamic data (Jones & Rowland‐Yeo, 2013; Peters, 2012).

As AAI has a high‐binding affinity to protein (Dickman et al., 2011), this leads to the differences in the free fraction of AAI in vitro, where medium without serum was used, as compared with the in vivo situation, where high protein levels are present (Blaauboer, 2010; Gülden, Dierickx, & Seibert, 2006). We have also measured the DNA adduct formation in cells exposed in the presence of fetal calf serum and the results confirm that protein binding significantly decreases the DNA adduct formation (data not shown) and should thus be taken into account. A correction for difference in free fraction between the in vitro and in vivo situation was included by multiplying the in vitro concentrations, obtained in absence of proteins, with a correction factor that amounted to 4.6. This factor was taken from Dickman et al. (2011), who showed that the free fraction of AAI in conditions resembling plasma is about 4.6 times lower than the free fraction in vitro in culture conditions where no proteins are added (Dickman et al., 2011).

Based on these assumptions the following equation was used to describe the formation of dA‐AAI and dG‐AAI adducts as a function of the AUC of the AAI venous blood concentration in the kidney in the PBK model:

(2) $$\mathrm{DNA}=A\times {\mathrm{AUC}}_{\mathrm{AAI}}\;$$

“*DNA*” is the amount of DNA adducts (number of adducts/10^8^ nt) formed, “*A*” is the slope, calculated based on the data from the in vitro experiments in which the in vitro AUC values were plotted against the amounts of DNA adducts that are formed within the in vitro experiment, measured in the absence of albumin (see Section 3). The in vitro AUC values were multiplied by 4.6 to account for the differences in free fraction between the in vitro and in vivo situation. “*AUC*_*AAI*_” represents the AUC of the AAI venous blood concentration in the kidney (CVK), defined as the total kidney concentration (CK) divided by the kidney:plasma partition coefficient (P_k:p_).

The PBK model allows estimation of the DNA adduct formation with different oral doses of AAI. The prediction of DNA adduct formation based on the AUC approach has been done before in other PBK or dynamic modeling‐based predictions (Paini et al., 2010). Based on the current state‐of‐the‐art, predictions on DNA adduct formation were made for the kidney as a whole. Yet, for some of the in vivo data used for evaluations, DNA adduct levels were reported for specific regions of the kidney and specific occurrence of DNA adducts might occur. This may lead to a source of uncertainty in the model predictions as indicated in Section 4.

### 2.8 Evaluation of the physiologically based kinetic modeling‐based reverse dosimetry approach to predict in vivo DNA adduct formation

To evaluate the potential of the in vitro‐in silico approach to obtain a dose‐response curve for in vivo DNA adduct formation of AAI, the DNA adduct formation predicted by the PBK modeling‐based reverse dosimetry approach was compared with in vivo data on DNA adduct formation in rat (Bieler et al., 1997; Chan et al., 2008; Dong et al., 2006; Mei et al., 2006; Pfau et al., 1990a), mouse (Arlt et al., 2011; Shibutani et al., 2007; Yun et al., 2012) and human kidney (Bieler et al., 1997; Nortier et al., 2000) available from the literature. Given the resistance of dA‐AA1 adducts towards repair (Geacintov & Broyde, 2017; Sidorenko et al., 2012), the reported in vivo DNA adduct levels in the different literature studies are considered the result of a linear increase in DNA adduct formation over time. Dividing the observed number of adducts by the duration of the experiment (in days) gives then an indication of the number of adducts that are formed per 24 hours. For evaluation of the in vitro‐in silico approach, these daily levels of DNA adduct formation were calculated for each study and compared with the in vitro‐in silico predicted daily DNA adduct formation (based on 24 hours in vitro incubations).

## 3 RESULTS

### 3.1 In vitro DNA adduct formation data

**FIGURE 2:**
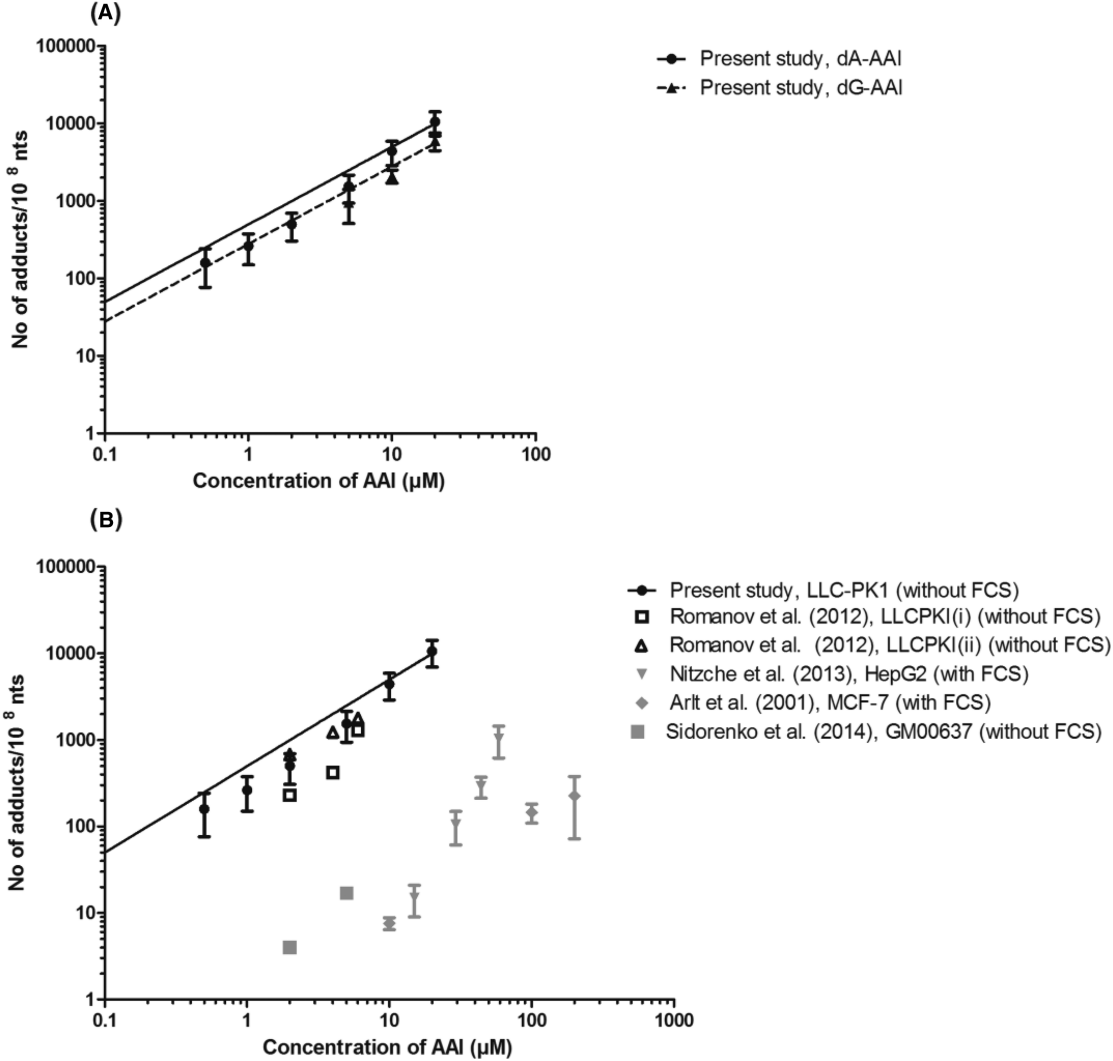
Concentration‐response curves for dA‐AAI (circle) and dG‐AAI adduct formation (triangle) in LLC‐PK1 cells upon 24 h exposure to increasing concentrations of AAI (μm). A, Expressed in number of adducts/10^8^ nt as quantified by LC‐ESI‐MS/MS (mean ± SD). B, Comparison with concentration‐response curves reported in literature quantified by ^32^P‐postlabeling. Black symbols show data from kidney cell lines and gray symbols show data from nonkidney cell lines. Linear equation was fit through the origin. AAI, aristolochic acid I; dA‐AAI, deoxyadenosine AAI; dG‐AAI, deoxyguanosine AAI; FCS, fetal calf serum; nts, nucleotides

Figure 2A shows the concentration‐response curve for AAI‐DNA adduct formation upon exposure of the LLC‐PK1 cells to increasing concentrations of AAI. The LC‐ESI‐MS/MS chromatogram for 543➔427 and 559➔443 transitions of hydrolyzed DNA isolated from LLC‐PK1 cells exposed to AAI shows that the dA‐ and dG‐AAI adducts eluted at 3.5 and 3.1 minutes respectively. These experimental data show a concentration‐dependent increase in both dA‐AAI and dG‐AAI DNA adduct formation at increasing concentrations of AAI up to 20 μm (the highest concentration tested). At 20 μm of AAI, the level of dA‐AAI DNA adducts formed after 24 hours of incubation resulted in 10 500 ± 3600 adducts/10^8^ nt (average ± SD of three independent experiments). The formation of dG‐AAI adducts was 1.8‐fold lower than the formation of dA‐AAI adducts (6000 ± 1500 adducts/10^8^ nt at 20 μm AAI). The results obtained reveal a linear relationship between the concentration and the level of adduct formation observed; dA‐AAI = 497.5 × [AAI] (*r* ^2^ = 0.98) and dG‐AAI = 277.5 × [AAI] (*r* ^2^ = 0.94). In line with literature data, dA‐AAI adducts are the major adducts formed, and because for these adducts most in vivo data are available, further analyses and PBK modeling‐based reverse dosimetry focused on dA‐AAI adduct formation.

Figure 2B shows the in vitro concentration‐response curve obtained in the present study for AAI concentration‐dependent dA‐AAI DNA adduct formation in the LLC‐PK1 cells as compared with in vitro concentration‐response curves reported in the literature. This comparison reveals comparable dA‐AAI DNA adduct levels detected in the present study with dA‐AAI DNA adduct levels detected before as reported in the studies available in the literature. It is also of interest to note that the results presented in Figure 2B indicate that DNA adduct formation reported so far is higher in kidney cell lines as compared with nonkidney cell lines.

### 3.2 Sensitivity analysis of the physiologically based kinetic models

Sensitivity analyses were performed at a low‐ and high‐dose level (0.1 and 100 mg/kg body weight of AAI) to identify the key parameters that influence the model outcome (AUC of the AAI venous blood concentration in the kidney). In both sensitivity analyses (Data S1B; see Supporting Information) the volume of the liver, biliary excretion and the partition coefficient of the liver were the most influential parameters in the PBK models for all three species, all expressing normalized sensitivity coefficients >0.1 (in absolute value). The sensitivity analyses also revealed that parameters related to the intestine (volume of the intestine, blood flow to intestine, S9 protein yield, the maximum rate of formation of AAIa metabolite and the Michaelis‐Menten constant for formation of AAIa metabolite) had a large influence on the model output only in the mouse PBK model, and that they were more influential at low oral dose levels than at high‐dose levels. In addition, the body weight in the mouse PBK model was a sensitive parameter at low oral dose levels.

### 3.3 Translation of the in vitro concentration‐response curve to in vivo dose‐response curves

**FIGURE 3:**
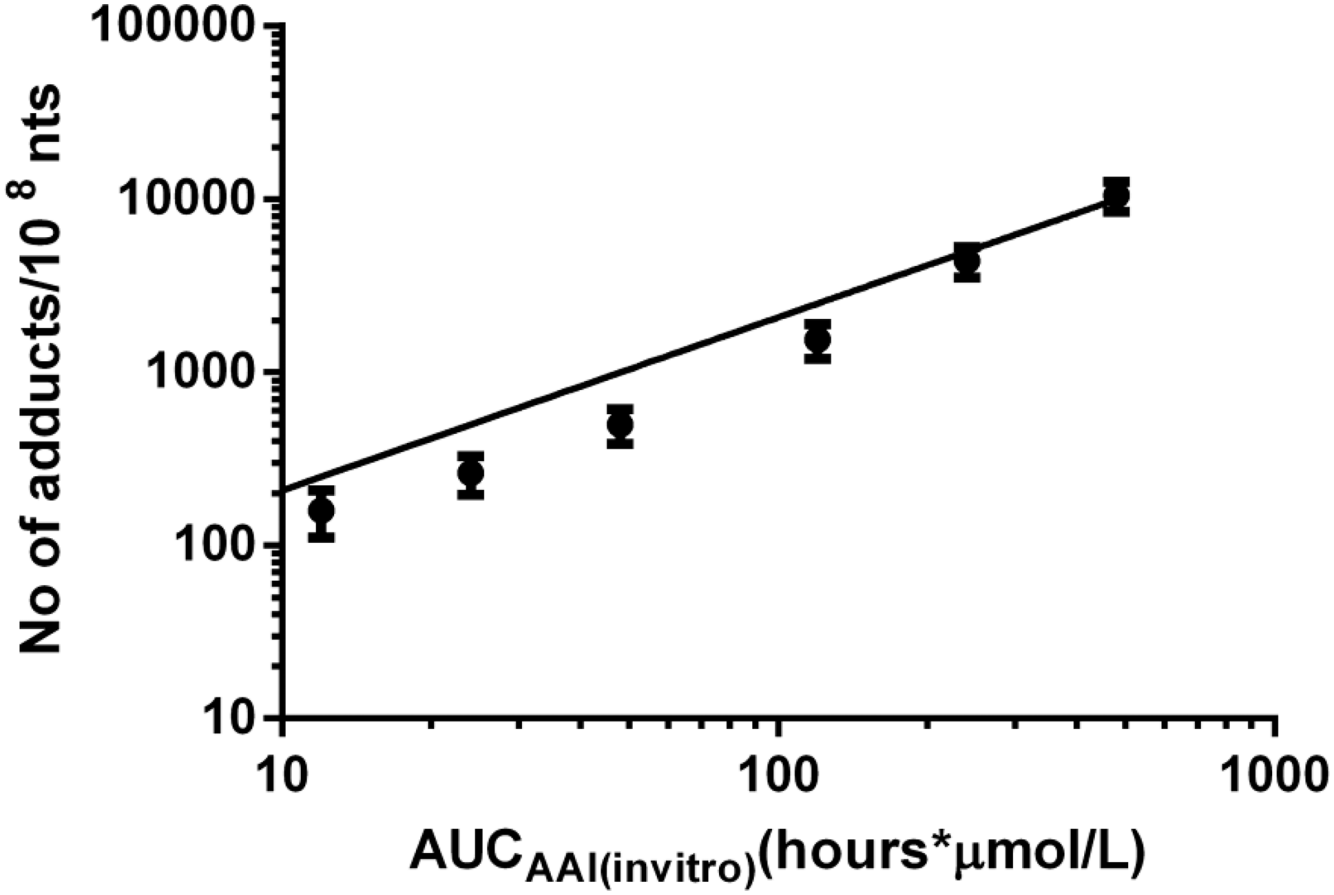
Deoxyadenosine‐AAI adduct formation in LLC‐PK1 kidney cell line expressed in number of adducts/10^8^ nt as a function of AUC_AAI(in vitro)_ (h × μmol/L), and quantified by liquid chromatography‐electrospray ionization‐tandem mass spectrometry (mean ± SD). Linear equation was fit through the origin. AAI, aristolochic acid I; AUC, area under the curve; nts, nucleotides

We assumed that the AUC and not the *C*_max_ is the most appropriate dose metric related to AAI‐induced DNA adduct formation because DNA adduct formation will depend more on cumulative exposure than on the maximum exposure concentration in the tissue of interest (Turteltaub & Dingley, 1998). Therefore, the concentration‐response curve of dA‐AAI adduct formation (Figure 2A), was converted to an AUC_AAI(in vitro)_ response curve (Figure 3) by multiplying the concentration by the time of incubation (24 hours). The AUC_AAI(in vitro)_ response curve presented in Figure 3 can be described by a linear equation through the origin by:

(3) $${\mathrm{DNA}}_{\mathrm{dA}}=20.7\times {\mathrm{AUC}}_{\mathrm{AAI}}$$

in which *DNA*_dA_ represents the amount of dA‐AAI DNA adducts (number of adducts/10^8^ nt) formed in the kidney cells at a certain *AUC*_AAI_ (h × μmol/L) of AAI. A correction factor for protein binding was applied to this in vitro concentration‐response equation (see Section 2) to account for the differences in free fraction between the in vitro and in vivo situation. When including this correction, the following equation was obtained:

(4) $${\mathrm{DNA}}_{\mathrm{dA}}=4.5\times {\mathrm{AUC}}_{\mathrm{AAI}}$$

This equation was incorporated in the PBK models, by defining that the corrected AUC of AAI in vitro should equal the AUC of the AAI venous blood in the kidney in the PBK model (see Equation 2 in Section 2), thus providing a link between the PBK model and the equation for DNA adduct formation in vitro and defining a PBK model that can predict DNA adduct formation as a function of the AAI dose. Given that the curves were defined in LLC‐PK1 cells, repair of the DNA adduct formation in the exposed cells is intrinsically taken into account (Li et al., 2006; Vamvakas, Dekant, & Henschler, 1989). Thus, the current equation implicitly represents both formation of DNA adducts and potential repair.

**FIGURE 4:**
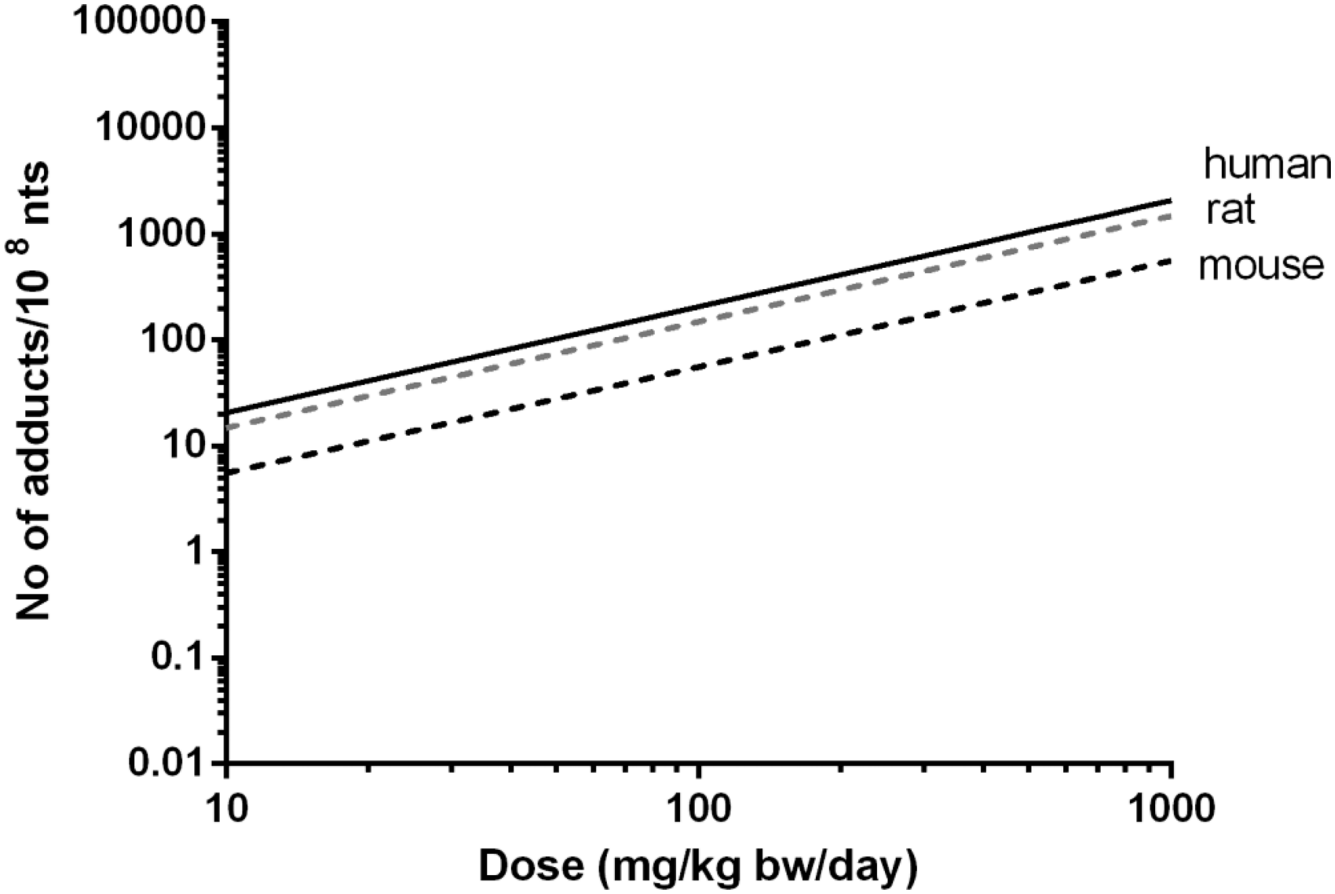
PBK modeling‐based reverse dosimetry predicted in vivo dose‐response curves for DNA adduct formation in the kidney of rat (gray dashed line), mouse (black dashed line) and human (black solid line). Formed adducts are presented as number of adducts per day. bw, body weight; nts, nucleotides

Figure 4 shows the predicted in vivo dose‐response curves for DNA binding of AAI in rat, mouse and human obtained by converting the in vitro AUC‐response curve for DNA adduct formation in LLC‐PK1 cells (Figure 3) by PBK modeling‐based reverse dosimetry. The predicted DNA adduct formation reveals that the species differences in kinetics result in DNA adduct formation being 1.4‐fold lower in rat kidney compared with human kidney and 3.7‐fold lower in mouse kidney than in human kidney at similar dose levels per kg body weight.

### 3.4 Evaluation of the in vitro physiologically based kinetic model‐based predictions of in vivo DNA adduct formation by aristolochic acid I in the kidney

**FIGURE 5:**
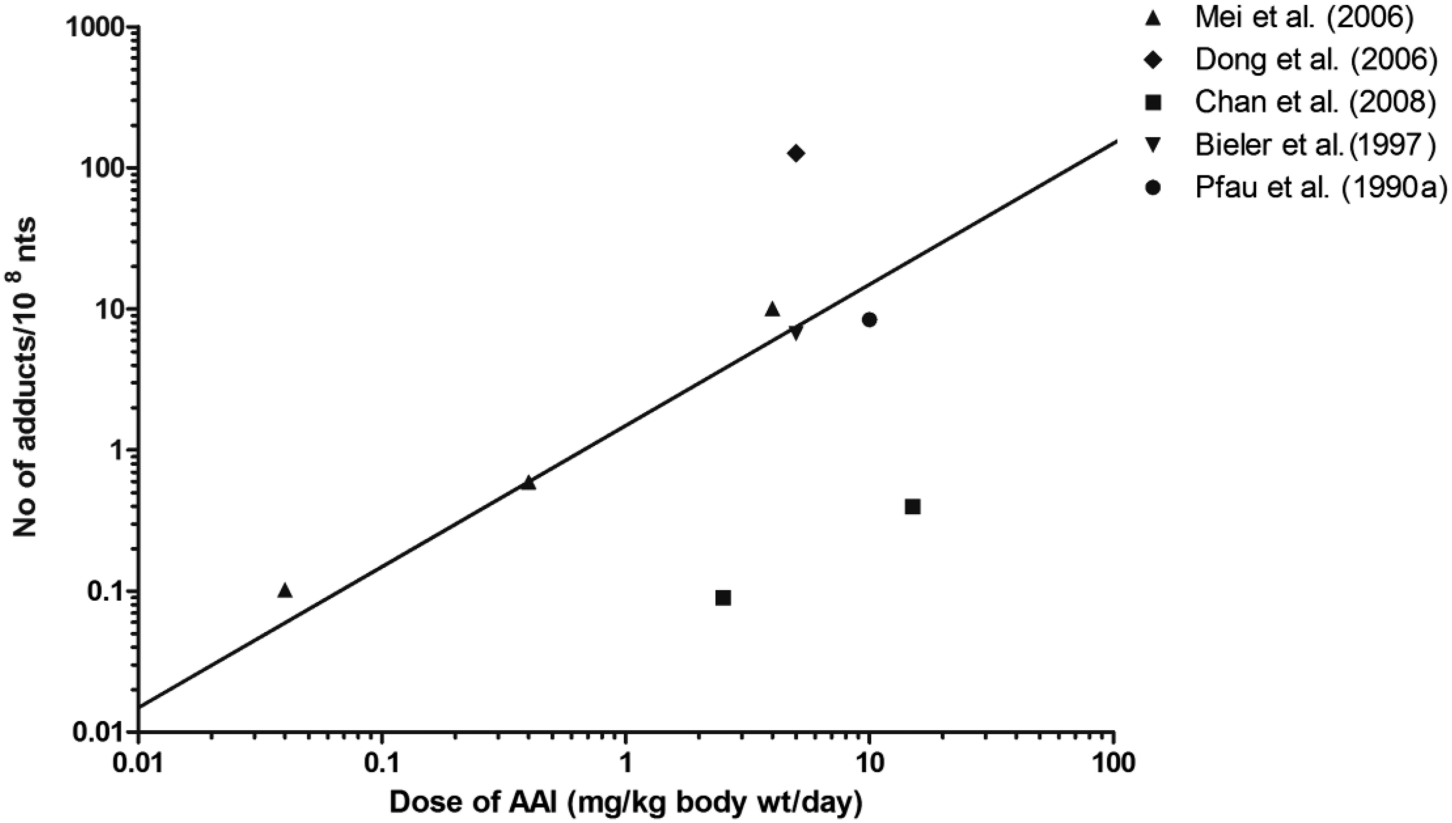
Comparison of PBK modeling‐based reverse dosimetry predicted dose‐dependent DNA adduct formation (straight line) in the kidney of rats exposed orally to AAI to data on in vivo AAI‐DNA adduct formation in the kidney of rats as obtained from the literature. Formed adducts are presented as number of adducts per day. See Table 1 for specifications of the experimental conditions for the in vivo studies. AAI, aristolochic acid I; wt, weight; nts, nucleotides

**FIGURE 6:**
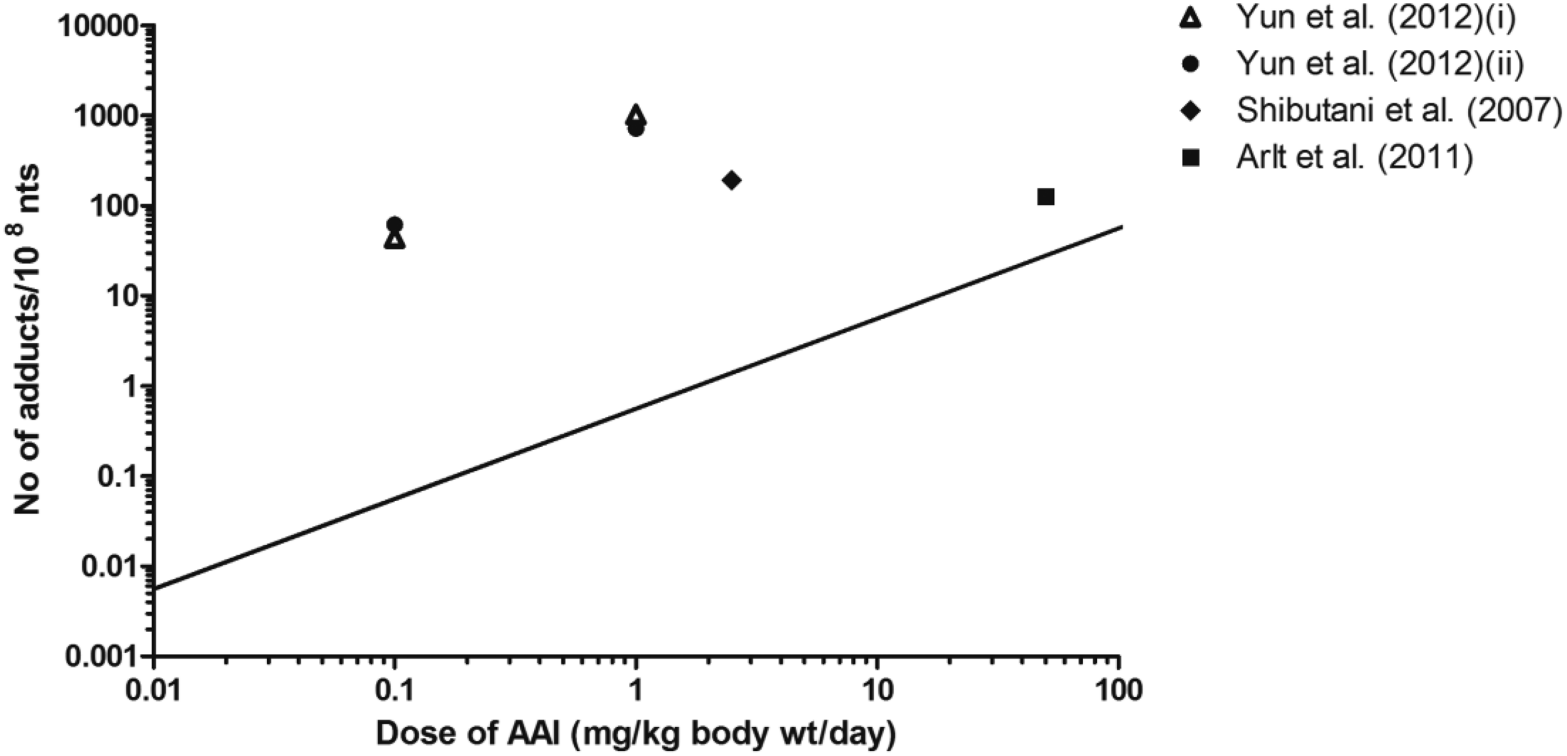
Comparison of PBK modeling‐based reverse dosimetry predicted dose‐dependent AAI‐DNA adduct formation (straight line) in the kidney of mice exposed orally to AAI to data on in vivo AAI‐DNA adduct formation in the kidney of mice as obtained from the literature. Formed adducts are presented as number of adducts per day. See Table 2 for specifications of the experimental conditions for the in vivo studies AAI, aristolochic acid I; wt, weight; nts, nucleotides

**FIGURE 7:**
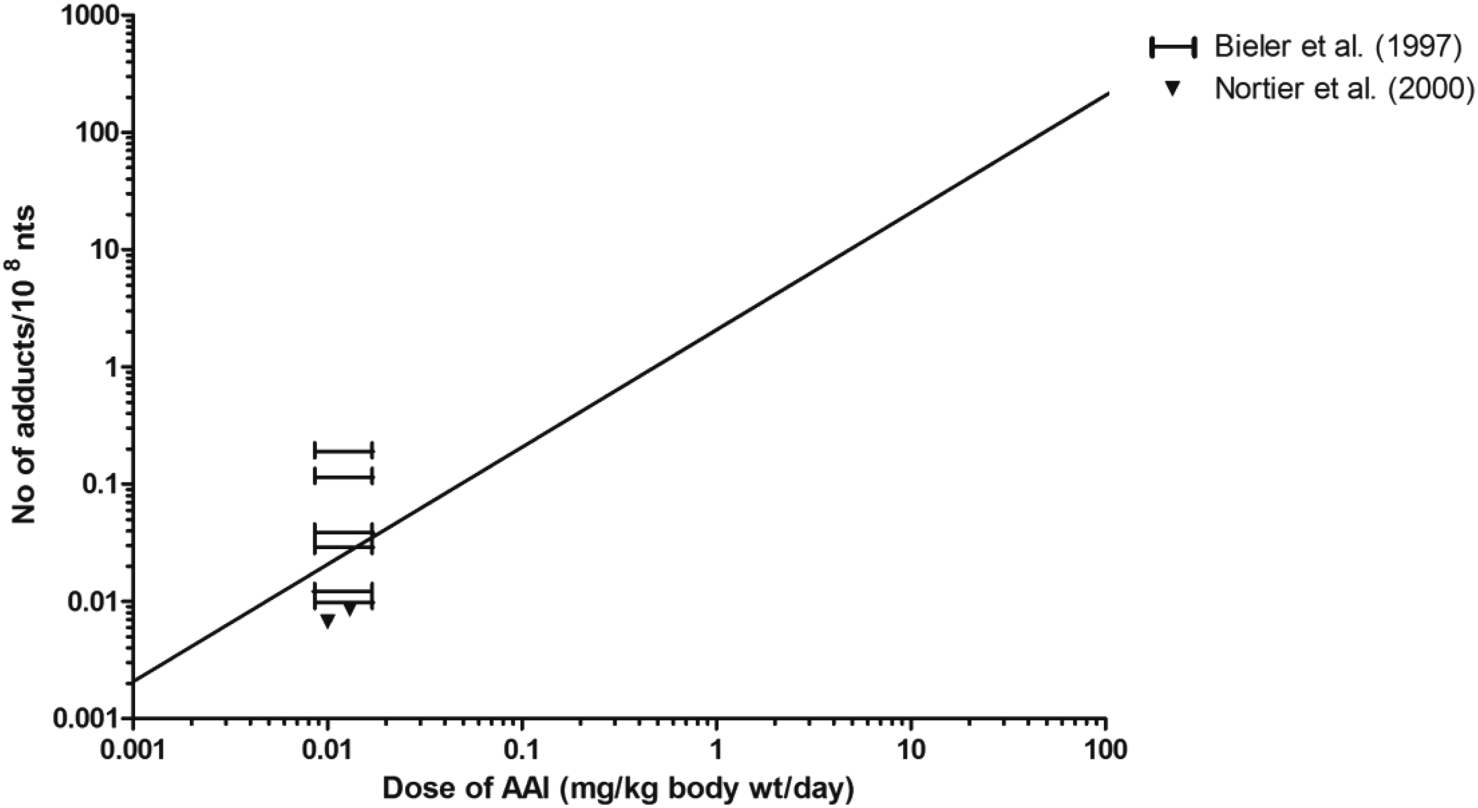
Comparison of PBK modeling‐based reverse dosimetry predicted dose‐dependent AAI‐DNA adduct formation in the kidney of humans (straight line) exposed orally to AAI to data on AAI‐DNA adduct formation in patients with AAN, as obtained from the literature. Formed adducts are presented as number of adducts per day. See Table 3 for specifications for the human studies, where the study from Bieler et al. (1997) presents data based on the estimated range of exposure from different individuals. AAI, aristolochic acid I; wt, weight; nts, nucleotides

To evaluate the outcomes of the in vitro PBK model based predictions for dose‐dependent AAI‐DNA adduct formation in the kidney, the predicted dose‐response curves for DNA adduct formation were compared with dose‐dependent DNA adduct formation in the kidney of rat, mouse and human as reported in the literature, taking into account the fact that the predictions were for a 0‐24‐hour time interval while the in vivo reported effects resulted from a variable number of days of exposure. Tables 1, 2 and 3 and Figures 5, 6 and 7 present an overview of in vivo literature data on DNA adduct formation in rat (Table 1 and Figure 5), mouse (Table 2 and Figure 6) and human (Table 3 and Figure 7) expressed as number of adducts per 10^8^ nt, and corrected to reflect the number of adducts per 10^8^ nt formed per day assuming accumulation of the adducts without substantial repair.

**TABLE 1:** DNA adduct formation in kidney of rats exposed to AAI as obtained from the literature

Species	Exposure route	AA composition	Dose^a^ (mg/kg body wt/day)	Exposure duration	Adduct type	No. of adducts/10^8^ nt^b^	Method	Figure	Reference
Rat	Oral	50% AAI	0, 2.5, 15^a^	1 day	dA	0, 0.09, 0.4	LC‐ESI/MS	5	Chan et al. (2008)
Rat	Oral	40% AAI	0, 0.04, 0.4, 4^a^	3 months	dA	0, 0.1, 0.6, 10.1	^32^P‐postlabeling	5	Mei et al. (2006)
Rat	Oral	AAI only	0, 5	7 days	dA	0, 126.6	^32^P‐postlabeling	5	Dong et al. (2006)
Rat	Oral	AAI only	0, 5	1 day	dA	0, 6.7	^32^P‐postlabeling	5	Bieler et al. (1997)
Rat	Oral	AAI only	0, 10	5 days	Total adducts (dA and dG)	0, 8.4	^32^P‐postlabeling	5	Pfau et al. (1990a)

^a^ Adjusted dose = dose × percentage of AAI.

^b^ Adjusted no. of adducts = no. of adducts/exposure duration (in days).

AAI, aristolochic acid I; dA‐AAI, deoxyadenosine AAI; dG‐AAI, deoxyguanosine AAI; LC‐ESI/MS, liquid chromatography‐electrospray ionization‐mass spectrometry.

**TABLE 2:** DNA adduct formation in kidney of mice exposed to AAI as obtained from the literature

Species	Exposure route	AA composition	Dose (mg/kg body wt/day)	Exposure duration (days)	Adduct type	No. of adducts/10^8^ nt^a^	Method	Figure	Reference
Mouse	IP	AAI only	0, 0.1, 1	1	dA	0, 43.9, 1020 (i)0, 61.6, 717 (ii)	UPLC‐ESI/MS (i) &^32^P‐postlabeling (ii)	6	Yun et al. (2012)
Mouse	Oral	AAI only	0, 2.5	9	dA	0, 191.1	^32^P‐postlabeling	6	Shibutani et al. (2007)
Mouse	Oral	AAI only	0, 50	1	Total adducts (dA and dG)	0, 125.7	^32^P‐postlabeling	6	Arlt et al. (2011)

^a^ Adjusted no. of adducts = no. of adducts/exposure duration (in days).

AA, aristolochic acid; AAI, aristolochic acid I; dA‐AAI, deoxyadenosine AAI; dG‐AAI, deoxyguanosine AAI; IP, intraperitoneal; UPLC‐ESI/MS, ultraperformance liquid chromatography‐electrospray ionization‐mass spectrometry.

**TABLE 3:** DNA adduct formation in kidney tissue of patients with aristolochic acid nephropathy as obtained from the literature

No. of case(s)	Exposure route	Age (year)/sex	Dose(mg/kg body wt/day) ^a^	Exposure duration (month)	Adduct type	No. of adducts/10^8^ nt^b^	Method	Figure	Reference
1	Oral	32/F	0.0086‐0.017	19	dA	0.030	^32^P‐postlabeling	7	Bieler et al. (1997)
1	Oral	28/F	0.0086‐0.017	13	dA	0.195	^32^P‐postlabeling	7	Bieler et al. (1997)
1	Oral	27/F	0.0086‐0.017	20	dA	0.118	^32^P‐postlabeling	7	Bieler et al. (1997)
1	Oral	42/F	0.0086‐0.017	21	dA	0.040	^32^P‐postlabeling	7	Bieler et al. (1997)
1	Oral	42/F	0.0086‐0.017	23	dA	0.010	^32^P‐postlabeling	7	Bieler et al. (1997)
1	Oral	56/F	0.0086‐0.017	19	dA	0.012	^32^P‐postlabeling	7	Bieler et al. (1997)
18	Oral	NA	0.01 ± 0.0014	15 ± 1.4	dA	0.007	^32^P‐postlabeling	7	Nortier et al. (2000)
19	Oral	NA	0.013 ± 0.0013	12 ± 1.1	dA	0.009	^32^P‐postlabeling	7	Nortier et al. (2000)

^a^ Dose is estimated based on the consumption of AAI by patients who took formula II, estimated to contain 2 mg/g of AAI from *Stefania tetranda* powder (Vanherweghem et al., 1993), three times a day and 70 kg body weight.

^b^ Adjusted no. of adducts = no. of adducts/exposure duration (in days).

AAI, aristolochic acid I; dA‐AAI, deoxyadenosine AAI; NA, not available; nt, nucleotide.

Figure 5 presents a comparison of the predicted dose‐dependent DNA adduct formation by the rat model as compared with the rat literature data. Our predicted values fall well within the range of the rat literature data. The results presented reveal that data from Bieler et al. (1997) and Mei et al. (2006) match well with our predicted DNA adduct values. Figure 6 presents a similar comparison for data from mice and reveals that our predicted DNA adduct levels were 4.5‐1800‐fold lower as compared with mouse literature data (Arlt et al., 2011; Shibutani et al., 2007; Yun et al., 2012).

Data reported for Belgian patients were used to evaluate the human model, although it should be kept in mind that there might be great uncertainty in the estimated dose levels. The uncertainties in intake estimates are often a reality in human data derived from intoxication incidents. Nonetheless, such intoxication incidents provide a valuable source of human data as experiments with defined dose levels and exposure regimens are, for ethical reasons, not allowed for a compound like AAI. As AA‐DNA adducts in human tissues show a long‐term persistence where 89 months after the discontinuation of exposure, levels of AA‐DNA adducts still being elevated above background (Nortier & Vanherweghem, 2002), a direct link with exposure might still be made. Figure 7 presents the dose‐dependent AAI‐DNA adduct formation predicted for human kidney and reveals that the DNA adduct levels reported for the human case studies fall within the range predicted for the levels of AAI‐DNA adducts accumulating in kidney tissue of patients with AAN.

## 4 DISCUSSION

The objective of the present study was to demonstrate whether PBK modeling‐based reverse dosimetry of in vitro concentration‐response curves for DNA adduct formation upon exposure to AAI could accurately predict in vivo dose‐response curves for AAI‐DNA adduct formation in the kidney of rat, mouse and human. The current study demonstrated that combining in vitro DNA adduct formation data with a PBK model for AAI kinetics is a promising approach to predict the DNA adduct formation in vivo.

In vitro DNA adduct formation was determined using LLC‐PK1 cells, which are proximal tubular cells from pig kidney that have been frequently used to assess in vitro AAI‐induced toxicity (Balachandran, Wei, Lin, Khan, & Pasco, 2005; Hsin et al., 2006; Romanov et al., 2012). Although AAI‐DNA adduct formation in pig cells may differ from AAI‐DNA adduct formation in kidney cells of rat, mouse and human, we predicted the DNA adduct levels for these three using this cell line. The LLC‐PK1 cell line is routinely used to study nephrotoxic effects of chemicals in humans because the cells exhibit many of the enzymatic and transport properties of human proximal tubule cells (Gstraunthaler, Pfaller, & Kotanko, 1985; Hull, Cherry, & Weaver, 1976), which are the cells that represent the direct target of AAI (Lebeau et al., 2005). Furthermore, comparison of the cytotoxicity of AAI in the LLC‐PK1 cells with the AAI cytotoxicity in primary renal human cells and even to the cytotoxicity in other cell types from other species described in the literature (Abdullah et al., 2016; Bastek et al., 2019; Huljic, Bruske, Pfitzenmaier, O'Brien, & Dietrich, 2008) reveals that species differences in dynamics of AAI toxicity may be limited. In addition, in our previous study, in vitro toxicity data on AAI in LLC‐PK1 cells provided adequate input for PBK model‐based prediction of in vivo kidney toxicity of AAI (Abdullah et al., 2016). Therefore, in the approach taken in the present study, species differences in AAI dynamics were assumed to be limited and species differences in kinetics were taken care of using species‐specific PBK models. Still, interspecies differences in cellular bioactivation of AAI may exist, which may be related to our consistent underprediction of dose‐dependent AAI‐DNA adduct levels in mice.

The present study revealed dA‐AAI to be the major adduct formed in vitro after exposure of LLC‐PK1 cells to AAI, which is in line with the major AAI‐induced DNA adduct formed in rat (Bieler et al., 1997; Stiborová et al., 1994), mouse (Shibutani et al., 2007; Yun et al., 2012) and human (Bieler et al., 1997). An overview of literature data revealed that the AAI‐DNA adduct formation in the LLC‐PK1 kidney cells was higher than that in HepG2 (human hepatoma) (Nitzsche, Melzig, & Arlt, 2013), MCF‐7 (human mammary carcinoma) (Arlt, Schmeiser, & Pfeifer, 2001) or GM00637 (human fibroblast) (Sidorenko et al., 2014) cells exposed at similar concentrations. It is interesting to note this clear difference in adduct levels when comparing cells from different organs (i.e., kidney vs. nonkidney), as these in vitro results are in line with the kidney being a target organ for AAI‐induced tumor formation (Arlt et al., 2002; Mengs & Stotzem, 1993). This tissue‐specific toxicity has been suggested because the capacity of the DNA repair processes in the kidney is lower than in other organs (Schmeiser et al., 1988) and/or for the ability of the proximal tubule cells to take up and concentrate AAs and their metabolites, making the kidney more susceptible to AA‐induced toxicity (Mei et al., 2006). In addition, the persistence of AA‐DNA adducts in the kidney tissue (Grollman et al., 2007; Nortier et al., 2000) may result from the resistance to repair and thus accumulation of adducts in the kidney. Together these arguments imply that the relatively higher sensitivity of kidney cells can be due to both toxicokinetic and toxicodynamic characteristics.

To translate the in vitro concentration‐response curve to in vivo dose‐response curves, PBK modeling‐based reverse dosimetry was used. To this end the previously developed PBK models for AAI kinetics in rat, mouse and human (Abdullah et al., 2016) were combined with in vitro data obtained in the present study for the AAI concentration‐dependent formation of the AAI‐DNA adducts in LLC‐PK1 cells. The in vivo dose‐response curves thus obtained for rat, mouse and human were compared with available data in the literature to evaluate the predictions. These literature data revealed large differences between different studies in the levels of kidney AAI‐DNA adduct formation at comparable dose levels. Rat data reported in the literature varied over three orders of magnitude, a difference that could only in part be explained by differences in the time of exposure during which the adducts could accumulate to their limited repair. In the case of the AAI‐DNA adduct, it has been shown that adducts are not efficiently repaired (Geacintov & Broyde, 2017; Sidorenko et al., 2012) particularly with the dA‐AA adducts, which are resistant to nucleotide excision repair mechanisms (Sidorenko et al., 2012). This observation thus also illustrates the variation that can be obtained between different in vivo studies reporting dose‐response behavior for the same endpoint. However, regarding effects in rats, data from Bieler et al. (1997) and Mei et al. (2006) match well with our predicted DNA adduct values. Regarding effects in mice, the PBK model‐based predictions for DNA adduct formation was at best 4.5‐fold lower than the literature data. Regarding effects in human, the predicted levels of AAI‐DNA adducts accumulating in kidney fall within the range reported for human patients with AAN.

The application of the quantification of DNA adducts in humans has been proposed to serve as an early indicator of cancer risks and can be used to evaluate species differences in risk assessment better. It is important to note that the presence of DNA adducts is considered a biomarker of exposure rather than a biomarker of effect (Neumann, 1986). This is because the majority of the DNA adducts may be nonmutagenic and/or may be repaired. In spite of the fact that DNA adduct formation is not a biomarker of effect, an increase in the level of DNA adducts is generally considered to be related to an increase in the risk of developing cancer (Paini et al., 2011).

Although the in vitro PBK modeling approach presented in the present study has uncertainties in its predictions due to assumptions in the parameters used in the model (Abdullah et al., 2016) and possible differences in toxicodynamics between the in vitro and the in vivo situation, the results of the present study show that these effects are likely to be limited for rats and humans; predictions made for the these species using the data from the LLC‐PK1 cells already quite adequately match the in vivo data for these species. The results reveal that the variation between different experimental studies reporting DNA adduct formation in the kidney in the same species appear to vary three orders of magnitude. The predicted level of AAI‐adduct formation in the kidney falls within this range and the difference between the predictions and the actual in vivo data is smaller than this variation between studies. Deviations observed between predicted and actually observed values may be due to several factors. These include the fact that at the present state‐of‐the‐art DNA adduct levels as well as the predictions made are directed at whole kidney tissue, while the actual formation of DNA adducts and tumors may vary between species in the different regions of the tissue (Cosyns et al., 1998). Another factor causing the deviations may be due to possible species differences in bioactivation of AAI to *N*‐hydroxyaristolactam I (Abdullah et al., 2016; Jadot et al., 2017; Stiborová, Frei, Wiessler, & Schmeiser, 2001), which was not covered in the in vitro DNA‐binding studies. Finally, experimental variations in DNA adduct level measurements, quantification techniques and uncertainties in exposure scenarios that occurred may also explain part of these deviations.

Based on the results obtained it can be concluded that the novel in vitro PBK modeling approach predicts DNA adduct formation within the ranges of reported in vivo data, indicating the potential of this approach to contribute to the reduction, refinement and replacement in animal testing.

## CONFLICT OF INTEREST

The authors have no conflict of interest to report.

## Supporting information


**Data S1.** Supporting Information
