# Supplementary material for: Defining in vivo dose‐response curves for kidney DNA adduct formation of aristolochic acid I in rat, mouse and human by an in vitro and physiologically based kinetic modeling approach
Source: J Appl Toxicol. 2020 Jul 7;40(12):1647–60. doi: 10.1002/jat.4024 (PMC7689901; doi:10.1002/jat.4024)
Supplement: Supplementary file 1 — Data S1. Supporting Information [file JAT-40-1647-s001.docx]

Supplementary data A. Mass balance equation and parameter specifications of PBK model for aristolochic acid I in the rat

**Compound abbreviation**

Aristolochic acid I AAI

Aristolochic acid Ia AAIa

**Compartment (Tissue (T)) abbreviation**

Small intestine I

Liver L

Kidney K

Slowly perfused tissue S

Richly perfused tissue R

Fat compartment F

Arterial A

Venous V

**Variable Unit abbreviation**

Blood flow rate to tissue lh^-1^ Q(T)

Cardiac output lh^-1^ QC

Concentration of AAI in tissue or blood µM C(T)_AAI_

Partitioning coefficient tissue:blood AAI - P(T) _AAI_

Volume of tissue or blood l V(T)

Amount AAI in tissue or blood µmol A(T) _AAI_

Maximum rate of formation metabolite, AAIa in tissue µmol h^-1^ Vmax(T)_AAIa_

Michaelis-Menten constant for formation metabolite,

AAIa in tissue µM Km(T) _AAIa_

Uptake rate AAI in intestine h^-1^ Ka

Excretion rate in bile h^-1^ Kbile

Excretion rate in urine h^-1^ Kurine

**Small intestine**

$$\frac{{dAI}_{AAI}}{dt}=\frac{{dUptake}_{AAI}}{dt} +QI*\left( {CA}_{AAI}-\frac{{CI}_{AAI}}{{PI}_{AAI}} \right)-\frac{{VmaxI}_{AAIa}*\frac{{CI}_{AAI}}{{PI}_{AAI}}}{{KmI}_{AAIa}+\frac{{CI}_{AAI}}{{PI}_{AAI}}}$$

**Uptake of AAI from GI tract**

$$\frac{{dUptake}_{AAI}}{dt}=-\frac{{dAGI}_{AAI}}{dt}=ka*{AGI}_{AAI}$$

$${AGI}_{AAI} \left( 0 \right)=Oral dose$$

$${CI}_{AAI}=\frac{{AI}_{AAI}}{VI}$$

**Liver compartment**

$$\frac{{dAL}_{AAI}}{dt}=QL*CA+QI*\frac{{CI}_{AAI}}{{PI}_{AAI}}-\left( QL+QI \right)*\frac{{CL}_{AAI}}{{PL}_{AAI}}- \frac{{VmaxL}_{AAIa}*\frac{{CL}_{AAI}}{{PL}_{AAI}}}{{Km}_{AAIa}+\frac{{CL}_{AAI}}{{PL}_{AAI}}}-\frac{{dEb}_{AAI}}{dt}$$

$${CL}_{AAI}=\frac{{AL}_{AAI}}{VL}$$

**Excretion rate in bile**

$$\frac{{dEb}_{AAI}}{dt}=Kbile*AL$$

$$Eb\left( 0 \right)=0$$

**Kidney compartment**

$$\frac{{dAK}_{AAI}}{dt}=QK*({CA}_{AAI}-\frac{{CK}_{AAI}}{{PK}_{AAI}})-\frac{{dEu}_{AAI}}{dt}$$

$${CK}_{AAI}=\frac{{AK}_{AAI}}{VK}$$

$${CVK}_{AAI}=\frac{{CK}_{AAI}}{{PK}_{AAI}}$$

$$\frac{{dAUCVK}_{AAI}}{dt}={CVK}_{AAI}$$

**Excretion rate in urine**

$$\frac{{dEu}_{AAI}}{dt}=Kurine*AK$$

$$Eu\left( 0 \right)=0$$

**DNA adduct formation**

$$DNA=A*{AUC}_{AAI}$$

‘DNA’ is the amount of DNA adducts (number of adducts/10^8^ nts) formed due to specific binding of AAI to either deoxyadenosine nucleosides or deoxyguanosine nucleosides at a certain ‘AUC_AAI_’ (hours*µmol/L) of AAI, and ‘A’ is the slope, respectively, calculated based on the data from the in vitro experiments.

**Slowly perfused tissue**

$$\frac{{dAS}_{AAI}}{dt}=QS*({CA}_{AAI}-\frac{{CS}_{AAI}}{{PS}_{AAI}})$$

$${CS}_{AAI}=\frac{{AS}_{AAI}}{VS}$$

**Richly perfused tissue**

$$\frac{{dAR}_{AAI}}{dt}=QR*({CA}_{AAI}-\frac{{CR}_{AAI}}{{PR}_{AAI}})$$

$${CR}_{AAI}=\frac{{AR}_{AAI}}{VR}$$

**Fat compartment**

$$\frac{{dAF}_{AAI}}{dt}=QF*({CA}_{AAI}-\frac{{CF}_{AAI}}{{PF}_{AAI}})$$

$${CF}_{AAI}=\frac{{AF}_{AAI}}{VF}$$

**Arterial blood compartment**

$${CA}_{AAI}={CV}_{AAI}$$

**Venous blood compartment**

$$\frac{{dAV}_{AAI}}{dt}=\left( QF*\frac{{CF}_{AAI}}{{PF}_{AAI}} \right)+\left( QK*\frac{{CK}_{AAI}}{{PK}_{AAI}} \right)+\left( QR*\frac{{CR}_{AAI}}{{PR}_{AAI}} \right)+\left( QS*\frac{{CS}_{AAI}}{{PS}_{AAI}} \right)+\left( QL+QI \right)*\frac{{CL}_{AAI}}{{PL}_{AAI}}-QC*{CV}_{AAI}$$

$${CV}_{AAI}=\frac{{AV}_{AAI}}{VV}$$

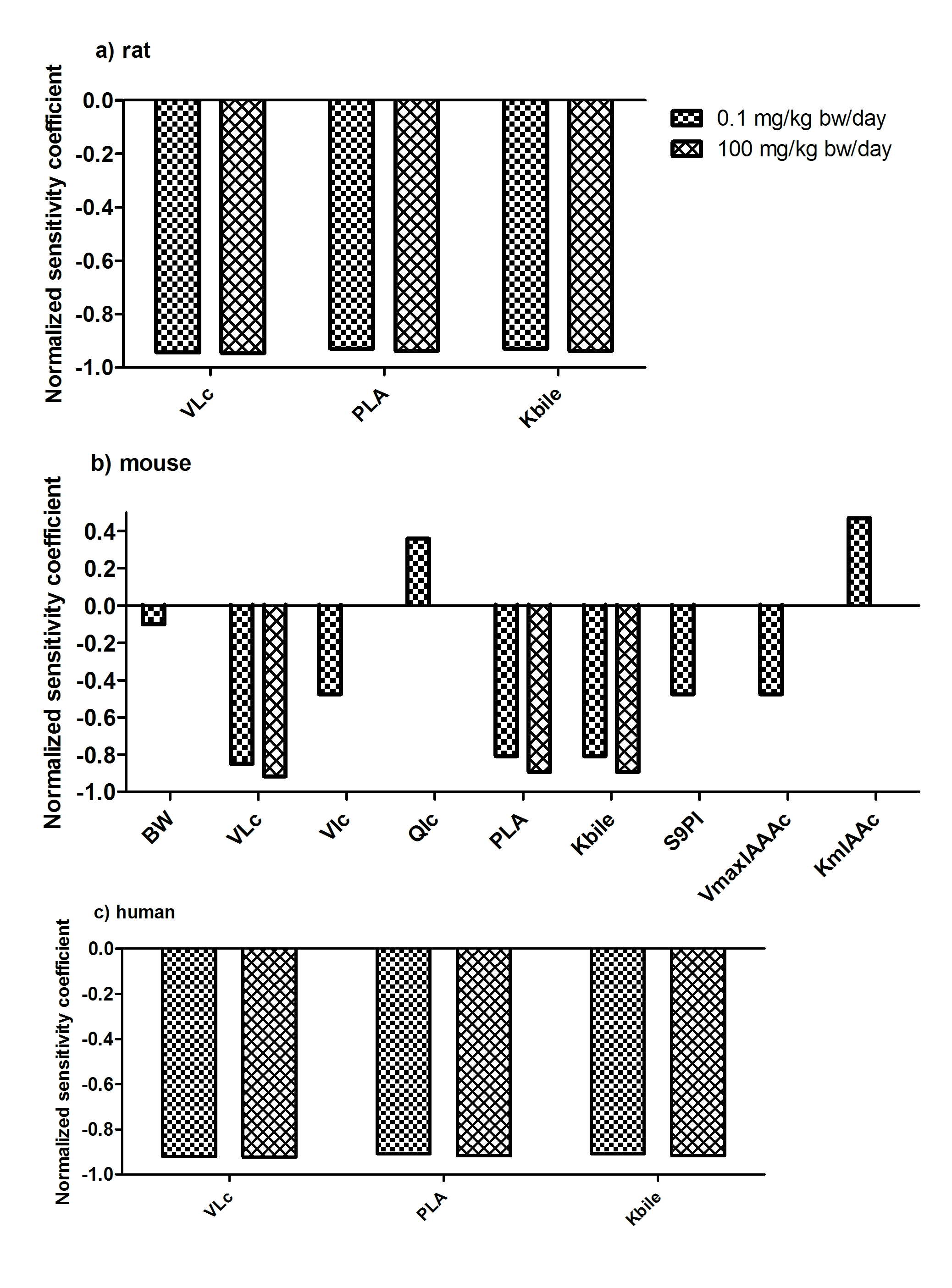


Supplementary data B. Normalized sensitivity coefficients for parameters of the PBK model for rat (a), mouse (b) and human (c) on AUC values in blood from single oral dose of 0.1 mg/kg bw and 100 mg/kg bw. Normalized sensitivity coefficients ≥ 0.1 are presented. VLc= volume of liver, VIc = volume of intestine, BW = body weight, QIC = blood flow to intestine, PLA = partition coefficient of liver, Kbile = excretion rate via bile constant, S9PI = intestinal S9 protein yield, VmaxIAAAc and KmIAAc = the maximum rate of formation and the Michaelis-Menten constant for formation of AAIa in intestine.
